# Case Report: A Case of Adult Methylmalonic Acidemia With Bilateral Cerebellar Lesions Caused by a New Mutation in *MMACHC* Gene

**DOI:** 10.3389/fneur.2022.935604

**Published:** 2022-07-05

**Authors:** Shengnan Wang, Xu Wang, Jianxin Xi, Wenzhuo Yang, Mingqin Zhu

**Affiliations:** ^1^Department of Neurology, The First Hospital of Jilin University, Changchun, China; ^2^Clinical College, Jilin University, Changchun, China

**Keywords:** cobalamin C deficiency, *MMACHC*, c.484G>A, methylmalonic acidemia and homocysteinemia, bilateral cerebellar lesions

## Abstract

Methylmalonic acidemia is a severe heterogeneous disorder of methylmalonate and cobalamin (Cbl; vitamin B12) metabolism with poor prognosis. Around 90% of reported patients with methylmalonic acidemia (MMA) are severe infantile early onset, while cases with late-onset MMA have been rarely reported. Few reported late-onset MMA patients presented with atypical clinical symptoms, therefore, often misdiagnosed if without family history. Herein, we report a 29-year-old female who was admitted to our hospital due to symptoms manifested as encephalitis. The brain MRI showed symmetrical bilateral cerebellar lesions with Gd enhancement. Laboratory tests showed significantly elevated levels of homocysteine and methylmalonic acid. A genetic analysis identified a novel homozygous mutation (c.484G>A; p.Gly162 Arg) in the *MMACHC* gene. The patient was diagnosed with MMA, and her symptoms improved dramatically with intramuscular adenosine cobalamin treatment. In conclusion, for patients with symmetrical lesions in the brain, the possibility of metabolic diseases should be considered, detailed medical and family history should be collected, and metabolic screening tests as well as gene tests are necessary for correct diagnosis. The mutation diversity in *MMACHC* gene is an important factor leading to the heterogeneity of clinical manifestations of patients with MMA.

## Introduction

Methylmalonic acidemia is an autosomal recessive genetic organic acid hematic disease due to methyl propylene acyl coenzyme A (MMCoA) mutase (methylmalonyl CoA mutase, MCM) defect or synthesis error in its coenzyme adenosyl cobalamin (AdoCbl) (1), causing abnormal accumulation of methylmalonic acid, propionic acid, and methyl citrate in the circulation, which can lead to pancreatitis, kidney failure, as well as neurological symptoms such as mental impairment, optic atrophy, and symptoms related to spinal cord and basal ganglia damage. Two main forms of the disease have been identified, including isolated methylmalonic acidurias and combined methylmalonic aciduria and homocystinuria. Isolated methylmalonic acidurias is due to defects of MCM or methyl malonyl CoA mutase (MUT) coenzyme AdoCbl, while combined methylmalonic aciduria and homocystinuria is characterized by elevated plasma homocysteine and decreased levels of the coenzymes AdoCbl and methyl cobalamin (MeCbl) (1). Cobalamin C deficiency (CblC) type is most common among all combined patients with methylmalonic aciduria and homocystinuria. The disease is caused by mutations in the MMACHC gene (OMIM ^*^609831) located on chromosome 1p34.1. The altered function of MMACHC results in a decreased intracellular production of adenosyl cobalamin and MeCbl, cofactors for the methyl malonyl-CoA mutase (EC 5.4.99.2) and methionine synthase (EC 1.16.1.8) enzymes. The deficient activity of these enzymes causes an elevation of methylmalonic acid and homocysteine and a decreased production of methionine. Symptoms of patients with CblC disease usually occur before 1 year old, while late-onset CblC disease often occur after 4 years old. The clinical classification of early-onset and late-onset CblC disease can correlate with the genotype of patients (2). To date, not more than 100 cases of late-onset methylmalonic acidemia (MMA) have been reported, with significant variations in clinical symptoms (3, 4). It has been proposed that the clinical heterogeneity of MMA is associated with the nature of different MMACHC mutations and the polymorphisms of other genes associated with cobalamin metabolism. Patients with late-onset MMA can be acute or insidious and show sudden deterioration. The clinical manifestations of late-onset patients are very heterogeneous and may be misdiagnosed if without family history. A recent study showed that the mean delay time from initial symptoms to diagnosis was 32.1 months (5). Herein, we report a 29-year-old female characterized by symmetrical bilateral cerebellar lesions, however, mainly manifested with symptoms of encephalitis. The patient was finally diagnosed with MMA, and her symptoms were improved dramatically with intramuscular hydroxocobalamin treatment.

## Case Report

**Figure 1:**
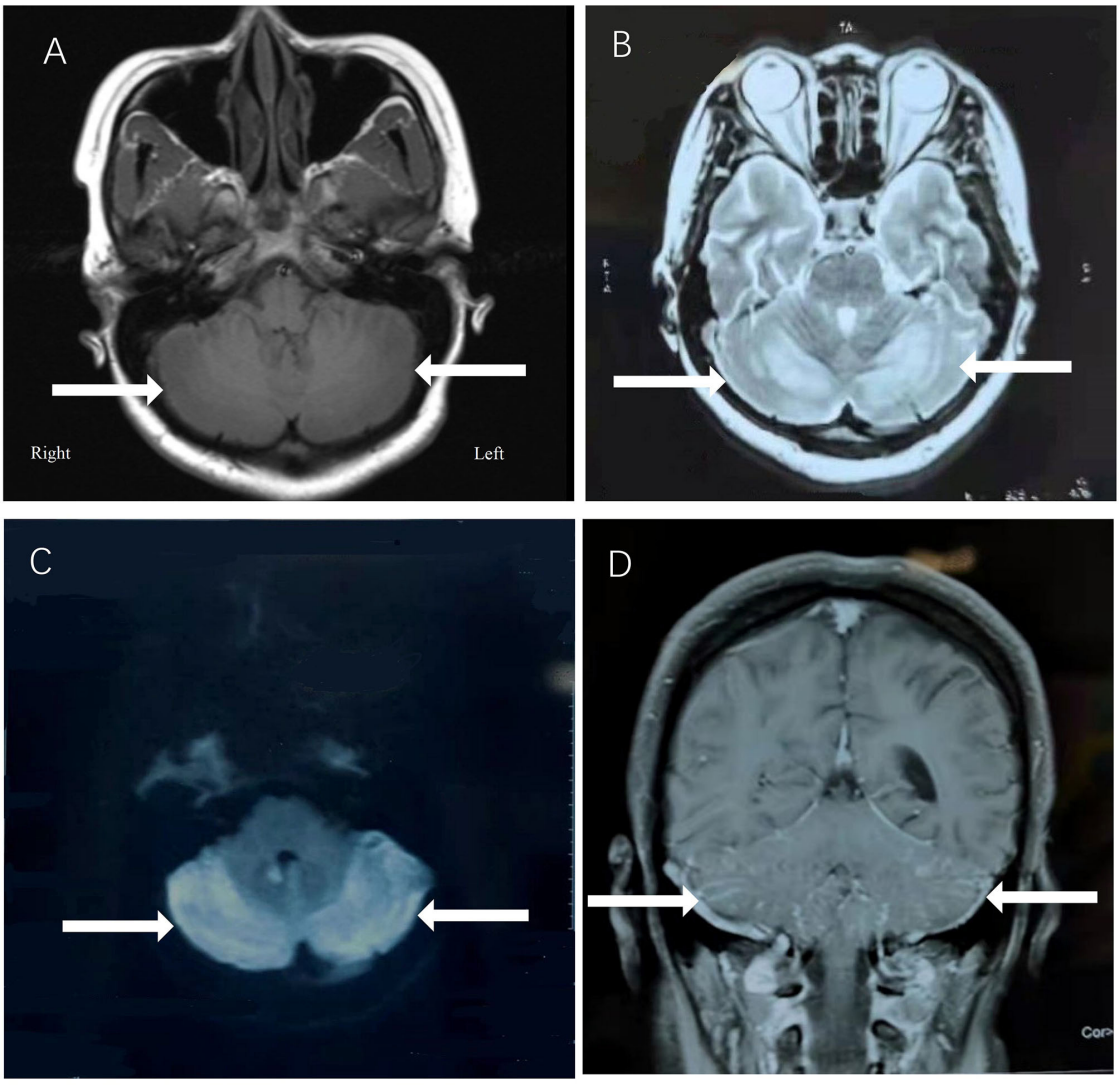
The patient's brain MRI showed abnormal signals in the vermis and in the bilateral cerebellar hemispheres, with low signal on T1-weighted image **(A)**, high signal on T2-weighted image **(B)**, and high signal on DWI sequence **(C)** with Gd enhancement **(D)**.

A 29-year-old female was hospitalized due to abnormal mental behavior for 10 days, mainly manifested as cognitive decline, cannot recognize family members, and inaccurate answers to questions. The patient had dizziness, headache, nausea, and vomiting occasionally; however, no fever was present during the disease course. The patient was diagnosed with hypothyroidism 1 year ago and was treated with oral levothyroxine sodium at 50 μg/day. A neurological exam showed delirium and increased muscle tone of limbs and ataxia. Meningeal signs (neck rigidity, Kernig's, Brudzinski's signs, jolt accentuation, and eyeball tenderness) were absent. All other neurological examination results were otherwise normal. Upon admission, based on the clinical symptoms, the possibility of encephalitis was considered. Methylprednisolone sodium was given at the dose of 1,000 mg/day for 3 days and penciclovir antiviral therapy was given as well. However, on the 4th day of admission, the patient began to show dysphoria at night, manifested as shouting and uncontrollable. A neurological examination showed increased muscle tone than before. The patient's brain MRI showed abnormal signals in the vermis and in the bilateral cerebellar hemispheres (Figure 1), with low signal on T1-weighted image (A), high signal on T2-weighted image (B), and high signal on DWI sequence (C) with Gd enhancement (D).

A laboratory test showed significantly elevated serum homocysteine (Hcy) levels (178.41 μmol/L, normal range 0–15 μmol/L). Serum folic acid levels (16.2 ng/ml, normal range 3.1–20.0 ng/ml) and serum vitamin B12 levels (248 pmol/l, normal range 139–652 pmol/l) were within the normal range. A thyroid function test showed increased levels of anti-thyroglobulin antibodies (163.45 IU/ml, normal range 0–4.11 IU/ml) and anti-thyroid peroxidase antibodies (>1,000.00 IU/ml, normal range 0–5.61 IU/ml); however, the thyroid hormone levels were within the normal range. Serum virus infection tests showed increased levels of herpes simplex virus (HSV) type I IgG antibodies (4.44 s/Co, normal range: <1.00), HSV type II IgG antibody (1.72 s/Co, normal range: <1.00), and Rubella virus IgG antibodies (2.76 s/Co, normal range: <1.00). A CSF routine test showed normal intracranial pressure (130 mmH_2_O, normal range: 80–180 mmH_2_O), slightly increased levels of IgG (47.03 mg/L, normal range 0–34.0 mg/L), the CSF cell count, and glucose and protein levels were within the normal range. The serum and CSF autoimmune encephalitis and paraneoplastic antibody panel tests were all negative. The patient's EEG and blood examination results were normal. Inflammatory markers such as *C*-reactive protein and procalcitonin levels were within the normal range. All the other immune function–related tests including antinuclear antibody; anti-deoxyribonuclease; complements; rheumatoid factor; anticardiolipin antibodies; antiphospholipid antibodies; lipoprotein A; factors II (prothrombin), VII, VIII, IX, XI, and XII; protein C; protein S; and antithrombin levels were normal.

**Figure 2:**
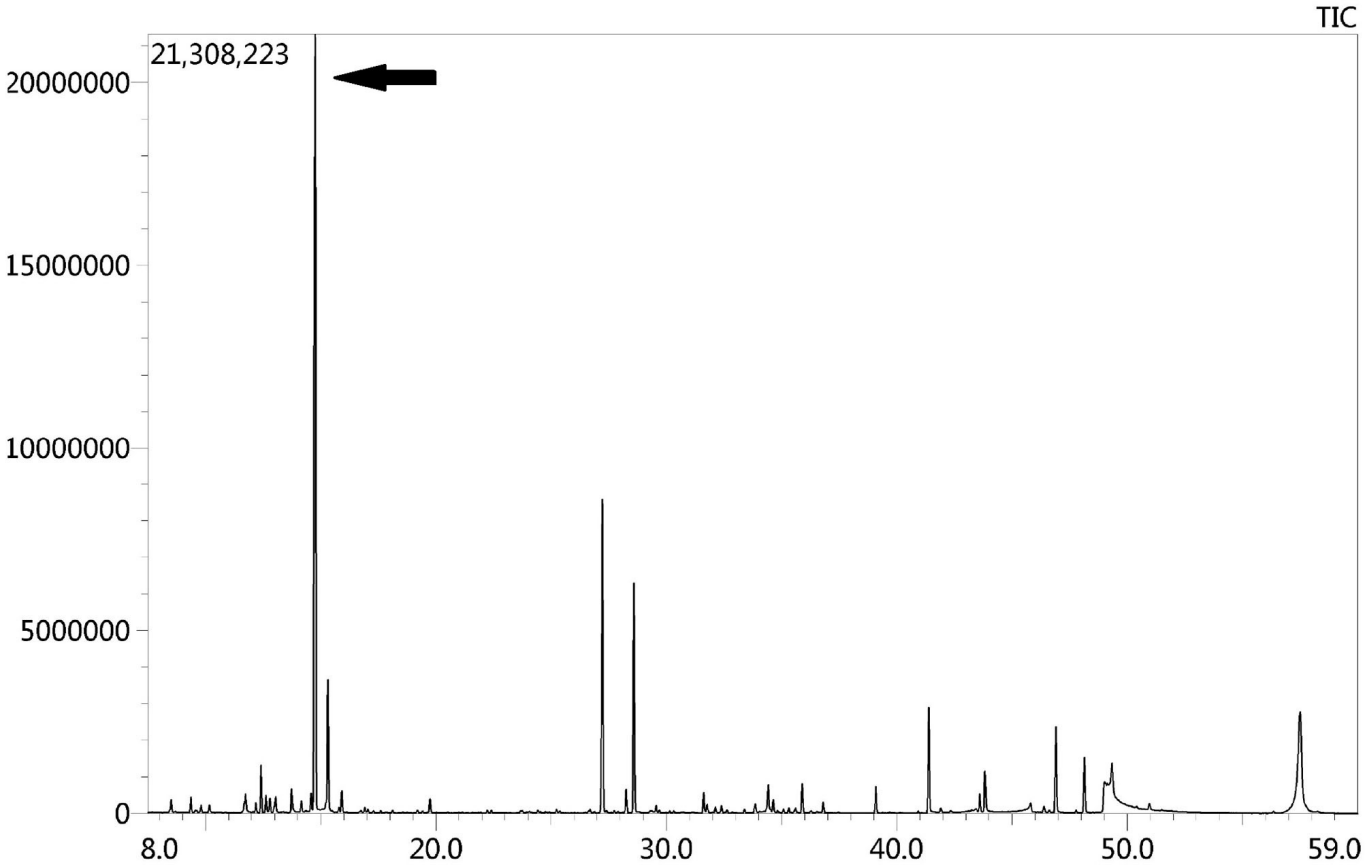
GC-MS urinary organic acid profile. The arrow showed spectrum of elevated methylmalonic acid, which was 100 times higher than normal levels.

**Figure 3:**
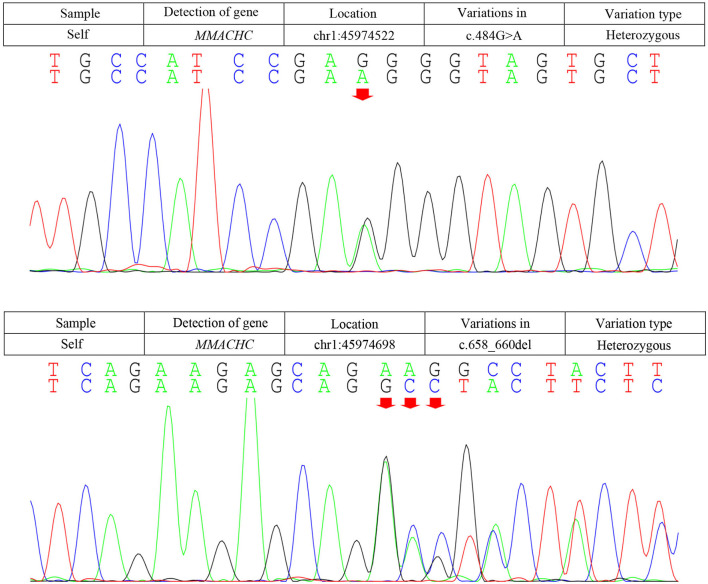
Results of the *MMACHC* gene test. Gene mutation in c.484G>A and c.658_660del. c.484G>A (red arrows) was observed due to an amino acid change in codon position 162, which is highly suspected to be a new genetic mutation.

Due to the symmetric lesions in the cerebellum showed by the brain MRI and significantly elevated serum homocysteine levels, metabolic disease was considered. Therefore, the blood and urine samples were sent out to Jilin Kingmed for Clinical Laboratory Co., Ltd. for a metabolic analysis. The results from blood examination showed that the ratio of propionyl carnitine and propionyl carnitine to acetylcholine increased, indicating MMA or propionic acidemia. Urine examination results showed increased methylmalonic acid (306.4 μmol/mmol creatinine, normal range 0.0–4.0 μmol/mmol creatinine) and methyl citrate (5.6 μmol/mmol creatinine, normal range 0.0–0.7 μmol/mmol creatinine) levels, indicating MMA (Figure 2); The levels of 3-hydroxypropionic acid and acetone were also found increased in the urea, indicating ketonuria. A genetic analysis was performed with the permission of the patient's parents and showed mutation in *MMACHC* gene: c.484G>A/c.658_660del (Figure 3). Therefore, MMA was diagnosed.

The patient was given adenosine cobalamin at 1.0 mg/day and l-carnitine at 1 g/day intramuscularly, and vitamin B6 10 mg/day and folic acid 15 ug/day were given orally as well. The patient's symptoms improved gradually, with decreased muscle tone and better cognitive status. The levels of homocysteine in the serum (from 178.41 to 35.42 umol/L) and the levels of methylmalonic acid in the urine were significantly decreased than before (from 306.4 to 6.1 μmol/mmol); the patient was discharged from the hospital on the 20th day of admission. After 2 months, the patient's cognitive ability had nearly returned to normal.

## Discussion

We present a 29-year female patient, who manifested an acute episode of cognitive impairment and psychiatric symptoms with symmetrical lesions in the cerebellum. Encephalitis was initially considered; glucocorticoids and antiviral therapy were given. However, the symptoms of the patient progressively deteriorated. Afterward, significantly increased levels of Hcy and methylmalonic acid were found, and mutation in *MMACHC* gene was detected; thus, late-onset CblC-type MMA was diagnosed.

The patient presented with acute cognitive impairment, psychiatric symptoms, and symmetrical abnormal signals in the cerebellum. Psychiatric symptoms such as aggression, irritability, and marked disturbance in sleep/wake cycles may occur in patients with autoimmune encephalitis (6), Moreover, anti-glutamic acid decarboxylase (anti-GAD)-associated bilateral symmetrical cerebellar lesions in patients with autoimmune encephalitis have also been reported (7). Thus, autoimmune encephalitis was initially considered and the patient was treated with glucocorticoids and antiviral therapy while awaiting CSF autoimmune antibody results. However, the patient's symptom deteriorated with the above treatment and CSF autoimmune antibody results came back negative. Therefore, the diagnosis of autoimmune encephalitis was excluded.

Most patients with MMA show varied symptoms in the infancy period, including poor feeding, hypotonia, seizures, coma, and death (8). The clinical features of late-onset CblC MMA are rarely reported and are quite different from those of early-onset ones (2). Cerebral white matter and basal ganglia lesions were the most common reported in early-onset patients, while cerebrum atrophy and white matter lesions were frequently reported in the late-onset patients (9). However, abnormal cerebellar signals were rarely reported with only three cases reported so far. Sheng et al. (10) reported a case of late-onset CblC MMA with subacute onset ataxia for 6 weeks. The T2-weighted brain MRI showed abnormally high signals in bilateral cerebellar hemispheres and right basal ganglia. Chang et al. (9) reported a case of late-onset CblC MMA with gait disturbance and psychiatric symptoms for a year. In this case, the brain MRI at the age of 15 showed altered signal from the dorsal portions of the cerebellar hemispheres. Wang et al. (11) reported another late-onset CblC MMA case with progressive cognitive impairment of 1-month duration, gait instability, bilateral upper and lower limb rigidity, urine incontinence, delirium, and auditory hallucinations. Bilateral abnormality of cerebellum cortex was found in diffusion-weighted imaging (DWI) and fluid attenuated in version recovery (FLAIR) sequence. The brain MRI of our patient upon admission also showed similar symmetrical bilateral cerebellar lesions with Gd enhancement. However, our patient mainly presented as cognitive impairment and psychiatric symptoms. Her EEG showed mild abnormalities. The abnormal mental behavior of the patient might be due to the excitatory toxicity, oxidative stress, and inhibition of methylation metabolism of Hcy (12). The characteristics of MMA cases reported in the previous literature along with our case were summarized in Table 1.

**Table 1:** The clinical presentations, treatments, and outcomes of cases with late-onset CblC deficiency.

**Patient No. [Reference]**	**Diagnose age**	**Clinical symptoms**	**MRI or EMG results**	**Serum Hcy levels (μM/L)**	**Gene mutations**	**Outcome**
1 (our patient)	29	Cognitive decline, cannot recognize family members, inaccurate answers and often non-sense	Symmetrical bilateral cerebellar lesions with Gd enhancement	178.41	c.484G>A/c.658_660del	Improved
2 (4)	11	Learning difficulties Behavioral changes Ataxia and myoclonic jerks	Dilation of subarachnoid Space frontoparietally	225	–	Improved
3 (13)	33	Insomnia, exaggerated Expression	Mild diffuse atrophy of cerebral cortex	65	c.482G>A/c.658_660del	Improved
4 (14)	19	Posture change	Hyperintensity in the cerebellum and right basal ganglia, with modest cerebrum atrophy	69.5	c.482G>A/c.445_446del	Improved
5 (13)	29	Irritability, euphoria Cognitive impairment	Mild diffuse atrophy of cerebral cortex	115.3	c.482G>A/c.658_660del	Improved
6 (10)	11	Clinical cognitive Deficit, limb weakness	Peripheral nerve damage	103.3	c.609G>A	–
7 (10)	18	Abnormal gait	Mild peripheral nerve damage	61.4	c.609G>A/ c.482G>A	–

Methylmalonic acidemia is a metabolic disorder of vitamin B12; the CblC-type affects the synthesis of adenosine cobalamin and mecobalamin, which catalyzes the conversion of Hcy into methionine, resulting in MMA with hyper Hcy-induced MMA (12). Genetic analysis is the gold standard for the diagnosis of MMA. The responsible gene has been confirmed to be *MMACHC* gene located on chromosome 1P34.1, and more than 50 mutation types have been reported (2). The type of gene mutation reported in patients with late-onset MMA includes c.482G>A, c.347T>C, c.609G>A, c.394C>T, c.440G>C, etc. (1). The most common mutation types in patients with CblC type in China were c.609G>A and c.658_660delAAG, and c.609G>A accounted for 55.4% of the total number of mutation types (15). The pathogenic genes of our patient are c.658_660delAAG and c.484G>A. The mutation of C. 658_660delAAG was common in late-onset CblC disease (16); however, the pathogenicity of c.484G>A has not been reported so far, and we highly suspected that it is a new genetic mutation, which is speculated to be related to the clinical heterogeneity of the patient. This mutation was not found in the Human Genome Mutation Database (HGMD). Several types of mutations were found in codon 161, while our mutation causes an amino acid change in codon position 162. An *in silico* evaluation of this variation was done with the Mutation tester, Polyphen2, and SIFT databases, and all judged this variation as disease causing. The father of our patient was found to be heterozygous for this mutation (c.484G>A; p.Gly162Arg). SIFT, PolyPhen2, and Mutation Taster were used to predict the protein damage of the mutation, and the results indicated that it was likely to be a pathogenic protein.

The patient was treated with intramuscular injection of vitamin B12 and oral administration of l-carnitine and folic acid. Her laboratory, imaging results as well as clinical symptoms significantly improved. The CblC type is mostly well-responsive to vitamin B12; therefore, patients with CblC-type MMA should be treated with vitamin B12 as soon as possible (17). MMA is a treatable disease involving multisystem damage caused by metabolic abnormality, whose clinical manifestations overlap with other common diseases of the nervous system. In clinical practice, the possibility of MMA should be considered for patients with older onset age and abnormally increased Hcy levels. A screening of hematuria metabolism and a gene testing at an early stage are of great value for diagnosis (18).

In conclusion, for patients with symmetrical lesions in the brain, the possibility of metabolic diseases should be considered, detailed medical and family history should be collected, and metabolic screening tests as well as gene tests are necessary for correct diagnosis. The mutation diversity in *MMACHC* gene is an important factor leading to the heterogeneity of clinical manifestations of patients with MMA. We also emphasized the importance of early recognition of CblC deficiency and early treatment with hydroxocobalamin for a better prognosis of the disease.

## Data Availability Statement

The datasets presented in this article are not readily available because of ethical and privacy restrictions. Requests to access the datasets should be directed to the corresponding author/s.

## Ethics Statement

Written informed consent was obtained from the individual(s) for the publication of any potentially identifiable images or data included in this article.

## Author Contributions

SW drafted the article and contributed to editing and revision. XW, JX, and WY contributed to patient follow-up. MZ has substantively edited the manuscript. All authors have read and agreed to the final version of this manuscript.

## Funding

This study was supported by grants from the Science and Technology Planning Project of Jilin Province (No. 20180520110JH), from the outstanding Young Teacher Training Program of Jilin University, and from the Jilin Kingmed for Clinical Laboratory Co., Ltd.

## Conflict of Interest

This study received funding from the Science and Technology Planning Project of Jilin Province (No. 20180520110JH), the outstanding Young Teacher Training Program of Jilin University and from Jilin Kingmed for Clinical Laboratory Co., Ltd. The funders had the following involvement with the study: The funders paid for the blood and urine metabolic analysis and gene sequence analysis. The authors declare that the research was conducted in the absence of any commercial or financial relationships that could be construed as a potential conflict of interest.

## Publisher's Note

All claims expressed in this article are solely those of the authors and do not necessarily represent those of their affiliated organizations, or those of the publisher, the editors and the reviewers. Any product that may be evaluated in this article, or claim that may be made by its manufacturer, is not guaranteed or endorsed by the publisher.

## References

[1] Zhou X Cui Y Han J. Methylmalonic acidemia: current status and research priorities. Intractable Rare Dis Res. (2018) 7:73–8. 10.5582/irdr.2018.01026 29862147 PMC5982627

[2] Carrillo-Carrasco N Chandler RJ Venditti CP. Combined methylmalonic acidemia and homocystinuria, cblC type. I. Clinical presentations, diagnosis and management. J Inherit Metab Dis. (2012) 35:91–102. 10.1007/s10545-011-9364-y 21748409 PMC4219318

[3] Liu YR Ji YF Wang YL Zhang BA Fang GY Wang JT. Clinical analysis of late-onset methylmalonic acidaemia and homocystinuria, cblC type with a neuropsychiatric presentation. J Neurol Neurosurg Psychiatry. (2015) 86:472–5. 10.1136/jnnp-2014-308203 25091368

[4] Augoustides-Savvopoulou P Mylonas I Sewell AC Rosenblatt DS. Reversible dementia in an adolescent with cblC disease: clinical heterogeneity within the same family. J Inherit Metab Dis. (1999) 22:756–8. 10.1023/A:1005508620919 10472537

[5] Wang S-J Yan C-Z Liu Y-M Zhao Y-Y. Late-onset cobalamin C deficiency Chinese sibling patients with neuropsychiatric presentations. Metab Brain Dis. (2018) 33:829–35. 10.1007/s11011-018-0189-3 29374341

[6] Uy CE Binks S Irani SR. Autoimmune encephalitis: clinical spectrum and management. (2021) 21:412–23. 10.1136/practneurol-2020-002567 34108243 PMC8461404

[7] Emekli AS Parlak A Göcen NY Kürtüncü M. Anti-GAD associated post-infectious cerebellitis after COVID-19 infection. Neurol Sci. (2021) 42:3995–4002. 10.1007/s10072-021-05506-6 34328578 PMC8322110

[8] Huemer M Diodato D Martinelli D Olivieri G Blom H Gleich H. Phenotype, treatment practice and outcome in the cobalamin-dependent remethylation disorders and MTHFR deficiency: data from the E-HOD registry. J Inherited Metab Dis. (2019) 42:333–52. 10.1002/jimd.12041 30773687

[9] Chang KJ Zhao Z Shen R-H Bing Q Li N Guo X. Adolescent/adult-onset homocysteine remethylation disorders characterized by gait disturbance with/without psychiatric symptoms and cognitive decline: a series of seven cases. Neurol Sci. (2021) 42:1987–93. 10.1007/s10072-020-04756-0 33000330

[10] Wang SJ Yan C-Z Wen B Zhao Y-Y. Clinical feature and outcome of late-onset cobalamin C disease patients with neuropsychiatric presentations: a Chinese case series. Neuropsychiatr Dis Treat. (2019) 15:549–55. 10.2147/NDT.S196924 30863077 PMC6391119

[11] Wang XL Sun W Yang Y Jia J Li C. A clinical and gene analysis of late-onset combined methylmalonic aciduria and homocystinuria, cblC type, in China. J Neurol Sci. (2012) 318:155–9. 10.1016/j.jns.2012.04.012 22560872

[12] Martinelli D Dionisi VC Deodato F. Cobalamin C defect: natural history, pathophysiology, and treatment. J Inherit Metab Dis. (2013) 34:127–35. 10.1007/s10545-010-9161-z 20632110

[13] Wu L-Y An H Liu J Li J-Y Han Y Zhou A-H. Manic-depressive psychosis as the initial symptom in adult siblings with late-onset combined methylmalonic aciduria and homocystinemia, cobalamin C type. Chin Med J. (2017) 130:492–4. 10.4103/0366-6999.199826 28218226 PMC5324389

[14] Wang SJ Zhao YY Yan CZ. Reversible encephalopathy caused by an inborn error of cobalamin metabolism. Lancet. (2019). 393:e29. 10.1016/S0140-6736(19)30043-1 30782345

[15] Fei W Han L Yang Y Gu X Ye J Qiu W. Clinical, biochemical, and molecular analysis of combined methylmalonic acidemia and hyperhomocysteinemia (cblC type) in China. J Inherit Metab Dis. (2010) 33(Suppl. 3):S435–42. 10.1007/s10545-010-9217-0 20924684

[16] Hu S Mei S Liu N Kong X. Molecular genetic characterization of cblC defects in 126 pedigrees and prenatal genetic diagnosis of pedigrees with combined methylmalonic aciduria and homocystinuria. BMC Med Genet. (2018) 19:154. 10.1186/s12881-018-0666-x 30157807 PMC6116561

[17] Fraiser JL Venditti CP. Methylmalonic and propionic acidemias: clinical management update. Curr Opin Pediatr. (2016) 28:682–93. 10.1097/MOP.0000000000000422 27653704 PMC5393914

[18] Kalantari S Brezzi B Bracciamà V Barreca A Nozza P Vaisitti T. Adult-onset CblC deficiency: a challenging diagnosis involving different adult clinical specialists. Orphanet J Rare Dis. (2022) 17:33. 10.1186/s13023-022-02179-y 35109910 PMC8812048

